# Impact of Emotional Harassment on Firm’s Value

**DOI:** 10.3389/fpsyg.2018.02333

**Published:** 2018-12-04

**Authors:** Yun Hyeong Choi, Hee Jin Park, Seong-jin Choi

**Affiliations:** ^1^School of Business, Hanyang University, Seoul, South Korea; ^2^Accounting/Tax and Management Information Systems, Kyonggi University, Suwon, South Korea

**Keywords:** power trip, emotional harassment, conglomerate, market reaction, Korean-listed firms, event study, event system theory, emotional contagion

## Abstract

The activities and consequences of workplace bullying and harassment have been widely explored in the literature but mainly studied within the scope of individuals or at the team level. Taking a holistic approach, we associate the concept of bullying with firm-level performance as well as stakeholders’ responses in the market. In this paper, we examine whether and how market investors react to the news of corporate harassment by top officials of publicly listed firms in Korea. Using a standard event study methodology and multiple regression analysis with matched sample, we find significantly negative stock price reactions to news of corporate bullying. We also find that the impact is more salient if emotional bullying is involved and discuss both the theoretical and practical implications of these findings.

## Introduction

Workplace bullying, defined as situations in which employees suffer from persistent and hostile interpersonal behavior at work (Leymann, 1996) has received growing attention. Extant studies in psychological and organizational research explore the negative impacts not only on the victim but also on other employees (Einarsen et al., 1994; Zapf et al., 1996; Mikkelsen and Einarsen, 2002), and firms have shown considerable interest in minimizing the harm of harassment within organizations (Langan-Fox et al., 2007). However, although previous empirical studies examined the relationship between bullying and its consequences at the individual and team level (e.g., Olweus, 1993; Einarsen, 1999; Einarsen et al., 2003; Griffin and O’Leary-Kelly, 2004; Hansen et al., 2006; Langan-Fox et al., 2007; Simons, 2008; Mathisen et al., 2008; Balducci et al., 2009; Nielsen et al., 2010; Dehue et al., 2012; D’Cruz et al., 2018), few have discussed the firm-level dynamics and consequences of bullying in detail. For instance, there have been frequent occurrences of bullying and crises in firms that cannot be solved at the micro level, such as the recent “nut rage” incident on Korean Air.^1^ Therefore, research on the corporate-level impact of bullying and its impact on a firm’s value, which have been seldom studied, should be conducted.

The term “bullying” was chosen for this research for a reason. Workplace bullying can be divided into abuse by the boss, the co-worker, or the subordinate (Einarsen and Mikkelsen, 2003). Top managers’ behaviors are closely related to the firm’s reputation and performance (Hambrick and Mason, 1984), and thus we focus on the bullying behaviors of TMT (Top Management Team) to identify the significance of the consequences at the level of the firm. Social undermining and abusive supervision behaviors of the heirs who acquire management rights or executives are different from bullying at work. Therefore, to more accurately define harassment by heirs and executives, we refer to assaults on subordinates as “power trips.” This expresses their behavior of harassing people and making their lives difficult just because they have more harassing most other employees. In fact, as a holistic approach, the concept of the power trip should be associated with firm-level performance as well as stakeholders’ responses in the market.

Moreover, to extend the prior research on workplace bullying, and especially the concept of the power trip, from the individual and organizational levels to the firm level, we consider two moderators—emotional harassment and conglomerate—in our regression model and attempt to apply them to psychological perspectives. First, we classify power trips into two categories: emotional harassment including both “verbal and physical” abuse, and non-emotional power trips. We hypothesize that not all power trip events are salient (Nigam and Ocasio, 2010) but depend on the components that constitute corporate harassment. According to Morgeson et al. (2015), the more disruptive and critical the event, the more likely it is to transmute or affect behaviors and other events. In addition, harmful talk stemming from negative emotions spreads more quickly than positive sentiments (Samson, 2006; Luo, 2009). Thus, we explore the negative interaction effects caused by emotional harassment on firms’ values. Second, and in the same vein, we also suggest that the impact of power trips does not affect firms’ value homogeneously; its consequences depend on the level of visibility. Visible firms tend to be subject to greater pressure to contribute more to corporate philanthropy than firms with lower visibility (Brammer and Millington, 2006; Gao and Hafsi, 2015). Moreover, if a renowned firm is the subject of an unexpected scandal, this quickly makes its way onto social media, such as Twitter and Facebook (Aula, 2010). Public anger that is rapidly spread through social media is soon reflected in market reactions. In short, most conglomerates operate in complex industry contexts and their visibility, created by the mass media, is higher than that of small firms. Therefore, the detrimental effect of power trips on conglomerates is much more salient for them than for less visible firms, such as SMEs.

This research provides an empirical contribution to the literature by extending the extant research on workplace bullying at the individual and team level to firm-level bullying through a simultaneous event study and regression analysis. The empirical findings in this study imply that firm-level power trips can be an important variable that affects the firm’s value because they undermine the firm’s credibility in the market. The current study also contributes to the literature on workplace bullying by applying and testing different management theories in the unique context of Korea. Event-oriented theories have been relatively rare, particularly when compared to feature-oriented theories (Morgeson et al., 2015). In addition, literature on the visibility of firms has been identified in the Korea context, and especially the *chaebol* environment. In the next section, we review the previous literature and develop our hypotheses. After detailing the methodology and analysis, the results are presented followed by the discussions of the study.

## Theoretical Framework

### Effect of the Firm-Level Power Trip on the Firm Value in the Market

The consequences of workplace bullying among employees and teams have been well documented. This brings about counterproductive workplace behaviors (Fox and Spector, 2005) and abusive supervision is related to organizational deviance, which puts organizations at risk (Robinson and Bennett, 1995). But, a precise definition for workplace bullying by top-tier managers was not clearly developed in the literature and this, then, is remained as a very broad, inclusive, elusive term (Bartlett and Bartlett, 2011). Bartlett and Bartlett (2011) categorized work-related bullying as following three types; workload, work process, and evaluation and advancement. However, these classifications are based on within-firm analysis. Therefore, we replace workplace bullying with the terminology of “power trip” to further clarify our firm-level interactions. Power trip is called *Gapjil* in Korea; this term has a negative connotation to it. It is neologism made by combining the word *Gap* which is used to introduce the first party in a contract as well as refers to a party’s superior and powerful status and *Jil*, a suffix for any actions that is negatively conducted. It is seemingly a similar concept, but these need to be distinct. Workplace bullying is based on hierarchy that reveals job position or relationships but, can even be caused by a subordinate. On the contrary, power trip can take place without being inside firm and the target may not be just employees. The motives of power trip often stem from wealth and social status in an authoritarian society. Korea has deeply been imbued with these thoughts which become a culture that has been followed by individuals. Therefore, an individual who was a victim in the past acts just like a perpetrator in the past when he or she is in the powerful position. As social malady, power trip in Korea is like a diving duck. It could be swimming on the surface or diving deep in the water, but whether it is seen or not, it is always there. This higher power (usually a manager or someone’s boss) tends to go to their head causing them to “power trip” and abuse their rights as a manager/boss/owner. Such as picking on people or making their lives difficult, “Just because they can.” is a person who is on a power trip. Bullying leadership is based on fear and control, but mainly on negative reinforcement and punishment. Negative reinforcement is an act required to avoid unpleasant and painful consequences, which forces employees to do their employers’ work as instructed. Such leadership creates an oppressive culture in the workplace. In this type of work culture, relationships among employees are based strictly on vertical relationships and form a hierarchical relationship where their superiors shape and control the individuals’ character. Employees lose self-esteem and self-confidence by succumbing in a servile fashion to the perpetrator’s threats and abuse. This can lower employee morale, undermine loyalty, and eventually reduce corporate productivity.

However, it is still questionable how markets react to the news of bullying by managers. This research provides two arguments concerning this issue. The first is based on the consumer’s perspective. The existing research suggests that consumers prefer goods that are ethically produced, and this may affect market reactions. In other words, the authors argue that a company’s good behaviors, which are reported by the mass media, create a general context for consumers’ evaluations. For example, Creyer and Ross (1997) find a significant correlation between consumers’ preference for a company’s service and products and the level of the company’s ethical performance. Murray and Vogel (1997) find that managers are more willing to purchase goods from ethical companies. Brown and Dacin (1997) find that consumers’ preferences for a new product are associated with consumers’ overall assessment of the company’s CSR (Corporate Social Responsibility) activities. On the contrary, consumers also experience negative emotions regarding companies’ unethical behaviors. For example, Choi and Lin (2009) find that consumers showed negative emotions such as anger, fear, worry, and disgust toward the Mattel Product crisis; they recalled which news was posted on online bulletin boards, and this led to a boycott campaign. In sum, prior research suggests that corporate ethnical behaviors unveiled by the mass media have a positive impact on consumers’ assessment or willingness to buy, while unethical activities can have a negative effect on consumers’ evaluations of products. The second argument is based on the investor’s perspective. A power trip not only affects consumers but also directly undermines investors’ confidence in the focal firm in a negative way. Kohut (1971) and Kernberg (1979) argue that power trips are a kind of narcissistic leadership caused by a mental disorder. The narcissism is defined as an inflated sense of self and privilege, grandiosity, and low empathy for others (O’Reilly et al., 2014); managers with this characteristic tend to make impulsive and risky decisions (Nevicka et al., 2011; Braun et al., 2015). Narcissistic leaders have a strong sense of self, but their self-concepts are unstable, so they are easy to anger when others make mistakes. In reference to a pathology, managers with narcissism tend to be more aggressive and more flawed (Bushman and Baumeister, 1998). Researchers in the upper echelon argue that characteristics of TMT can signal something about the organization’s legitimacy, which influences investors’ evaluations of the firm (Higgins and Gulati, 2006). The leads us to expect that managers exerting undue power over subordinates make narcissistic and risky decisions, and investors tend to undervalue this kind of firm; this premise yields the following hypothesis.

H1. The firm-level power trip has a negative effect on a firm’s value.

### The Moderating Effect of Emotional Harassment

As power trips can occur at the individual, team, and organizational level, they can be classified into many different types. An abusive person can exercise power and control over the victim through physical, psychological, sexual, or financial abuse. In this study, we focus on “emotional” harassment including verbal and non-verbal harassment. On the one hand, the behaviors that simply abuse social superiority in the relatively upper class have potentially abusive power, but no emotions are involved (e.g., push-driven distribution sales, unfair labor practice, and “tunneling”). On the other hand, according to Keashly (1997) and Lutgen-Sandvik (2003), “emotional abuse is the term to articulate the hostile verbal and non-verbal behaviors that are not explicitly tied to sexual or racial content yet are directed at gaining compliance from others.” For example, yelling or screaming, nagging, intimidation, aggressive eye contact, negative rumors, ridiculing someone in front of others, silent treatment, and emotional blackmail is examples of emotional abuse. Conceptually, the term “abuse” is cruel and violent treatment among people while the term “harassment” which is intended to trouble or annoy someone is persistent attacks and criticism causing worry and distress. In this respect, the latter word is more appropriate for this research because it is not on a one-off but persisting for the corporate heirs and executives to inflict physical and traumatic damage on their victims. Previous study finds that organizations must have proper processes and procedures to address workplace abuse because it is their financial self-interest to do as well as to protect their workers. (Koonin and Green, 2007). Existing studies on emotions have emphasized that they play an important role in interactions insofar as they concern attitudes, ideas, thoughts, and behaviors (Ekman, 1993; Côté, 2005; Kopelman and Rosette, 2008; Van Kleef et al., 2010). While the expression of negative emotions (e.g., anger, shame, guilty) generally makes social interactions difficult, expressing and amplifying positive emotions could be equally instrumental in shaping relationships with others as they facilitate goal attainment at work (Wong et al., 2013). Such effects are spread through what is called “emotional contagion” (Hatfield et al., 1993). Organizational and psychological researchers have investigated emotional contagion through a variety of field studies (Totterdell et al., 1998; Totterdell, 2000; Bartel and Saavedra, 2000; Barsade, 2002), which have shown that people respond heterogeneously to positive and negative emotions, while negative events are inclined to trigger stronger emotional and behavioral responses than positive or neutral events (Cacioppo et al., 1997). Barsade (2002) also found that unpleasant emotions tend to generate greater emotional contagion than pleasant ones, which is referred to as the ripple effect. Because anger is one of the most contagious emotions (Tavris, 1984; Mattila and Enz, 2002), anger derived from emotional harassment (e.g., abusive language and even assault) by a manager and revealed through media coverage will elicit a more intense response from consumers and investors than a non-emotional power trip. Decision-making is greatly affected by emotions (Luce, 1998; Ruth, 2001) and previous studies have explored how consumers use information on emotions to arrive at efficient decisions. Despite the importance of emotional contagion in making decisions, empirical research on emotional contagion is limited at the micro level. Thus, it is necessary to study how TMT events involving emotional harassment generate negative emotions among consumers and investors that affect a firm’s value. In addition, according to event system theory, not all events are homogeneous but tend to be heterogeneous (Morgeson et al., 2015). From this perspective, events can occur at any level, regardless of the individuals, teams, organizations, and environments involved. Thus, a strong individual event like a TMT’s power trip could explain relationships between the event and the firm’s value. Organizations confront numerous events every day; however, not all of them are influential (Nigam and Ocasio, 2010). Thus, each event could have different results depending on whether emotional harassment is involved. To identify whether emotional harassment is involved in firm-level power trips, event system theory focuses on the crucial event feature in terms of its novelty, disruption, and criticality (Morgeson et al., 2015). First, novelty describes the extent to which an event is distinctive in comparison to past events, and thus stands for a new phenomenon (Lee and Mitchell, 1994; Morgeson, 2005). Second, disruptive events may deviate from normal routines (Zellmer-Bruhn, 2003). Disruptive events destroy entities outside of their stereotyped way of thinking (Morgeson et al., 2015). Finally, criticality indicates the degree of importance of the event (Morgeson and DeRue, 2006) and typically leads to additional analyses and changes (Vaara, 2003). Power trips by authorities lead to greater public outrage via emotional contagion than non-emotional power trips. In the same vein, negative word of mouth conveying negative emotions spreads more quickly than positive sentiments (Samson, 2006; Luo, 2009). In short, emotional harassment is more destructive because it is novel, disruptive, and critical compared to events that are emotionally neutral.

H2. The impact of firm-level power trips on a firm’s value is negatively interacted when emotion harassment is involved.

### The Moderating Effect of Conglomerates

The importance of a firm’s visibility and celebrity status as a source of competitive advantage is well documented in the organizational literature (e.g., Hall, 1992). Previous studies have argued that a firm’s visibility and celebrity are related to the firm’s performance and legitimacy (Deephouse, 2000; Roberts and Dowling, 2002). Organizations with many subsidiaries and considerable revenue are likely to be more visible. The more the media monitor the firm’s behavior, the more likely their social activities are to be visible (Matten and Moon, 2008). According to Rindova et al. (2006) and Pfarrer et al. (2010), the firm’s visibility is increased when the media highlight a firm as a protagonist in the corrupted institution (Rindova et al., 2006). Examples of highly visible firms in our sample include conglomerates, and especially *chaebol* (in Korea). These *chaebols*’ unconventional and controversial actions have attracted media attention touting their distinctive cultures, charismatic leaders, and singular identities. In a country where workers are often expected to show unquestioning loyalty, cases have focused on immoral behavior by the rich and powerful, along with public indignation at the family-run conglomerates known as *chaebol*, which dominate South Korea’s economy.

Existing studies suggest that larger firms tend to be more visible and more likely to pay attention to social responsibility; they may also suffer from damage that leads to a bad reputation (Udayasankar, 2008). This argument is consistent with Saiia et al. (2003), who found that highly visible firms tend to make larger philanthropic gifts. Highly visible firms are also subject to more pressure and scrutiny from the public to take practical steps and meet social needs (Campbell and Slack, 2006). Korean *chaebols* have been criticized for misbehavior. Since they became the pivot of the Korean economy, Koreans have considered it a measure of social success to get a job at a *chaebol* conglomerate but, simultaneously, they hold anti-*chaebol* sentiments about misbehaviors and owner risk. Using the catchphrase ‘*chaebol* reformation’ to improve its transparency and accountability, they argue that the *chaebol* focuses on accumulating wealth and inheriting the company. Because firms with higher visibility are under great pressure to contribute more to activities involving social responsibility than firms with lower visibility (Gao and Hafsi, 2015), merely symbolic donations are unacceptable and may severely damage the firm’s reputation (Boiral, 2007; Lyon and Maxwell, 2011). As corporate philanthropy influences corporate perceptions for a variety of stakeholders, including investors, customers, suppliers, actual or potential employees, and the voluntary sector (Smith, 1994; Himmelstein, 1997; Saiia et al., 2003), a firm’s responsiveness to its stakeholder environment plays an important role in the firm’s value (Brammer and Millington, 2006). Chang (2003) conducted a study of 419 *chaebol* affiliates from 1986 to 1996 and found that major shareholders, who are also managers, can be valued by other shareholders. Thus, managers from a *chaebol* family who hold large numbers of shares tend to abuse the corporate power given by their ownership. These behaviors undermine the firm’s ethical and social responsibilities. In this context, when owners or top managers of Korean *chaebol* behave unethically, power trips are more negatively evaluated by the media and stakeholders. This reasoning leads us to expect that conglomerates with high visibility will experience more negative consequences when power trips occur.

H3. The impact of firm-level power trips on a firm’s value is negatively interacted for conglomerates.

## Sample Selection and Research Design

### Sample Selection

We selected companies that were reported to the media as culpable of a power trip from those listed on the Korea Stock Exchange. In this study, the power trip variable was set based on the date of the first report on the media such as newspaper and news. For example, the nut rage incident was an air rage incident that occurred on December 5, 2014, at John F. Kennedy International Airport in New York City. Korean Air vice president Heather Cho, dissatisfied with the way a flight attendant served nuts on the plane, ordered the aircraft to return to the gate before takeoff. As another example, On May 8, 2013, Namyang Co., was criticized following an accusation that the firm had pressed local franchises to buy products for a long time, as well as a tape-recording of rough words to local owners. The coverage of each case quickly spread through the media after December 5, 2014 and May 8, 2013.^2^ Since the news of power trip is rapidly spread through media such as SNS and news, the first news day was set as an event day and the stock return from day -1 to day +1 was observed based on the event day. The sample period is from 2013 to 2018. The information on these companies was collected through media press releases on the Internet and in newspapers. We obtained financial data from the KIS-VALUE, which provides the financial statements of all listed firms, and stock data from the Fn-Guide.

The sample selection process is summarized in Table 1. The sample used in the event study is a total of 31 firm-year observations. Specifically, there are 33 companies reported to the media as those said to be guilty of harassment. Among them, we conducted an event study on a total of 31 firm-year observations except for two companies belonging to KOSDAQ companies. Since 2015, there has been a significant increase in media coverage of corporate harassment and it is emerging as a social problem.

**Table 1:** Sample selection.

Panel A: Sample selection criteria							Sample
Companies reported to the media as guilty of power trips between 2013 and 2018							33
Less: KOSDAQ firms							(2)
Sample used in the event study							31
Less: A company reported to the press as a company guilty of a power trip in the same year							(3)
Less: Companies for which financial data cannot be obtained from the FN-Guide							(7)
Sample used for regression analysis							21

**Panel B: Number of companies reported to the media as companies guilty of power trips by year**							

	**2013**	**2014**	**2015**	**2016**	**2017**	**2018**	**Total**

*N*	1	1	7	6	7	9	31


There are cases where the same company is reported as guilty of harassment several times within one accounting period. When the same company is reported in this way, it is included in the event study analysis, but is used only as one sample in the OLS regression analysis. OLS regression analysis was carried out on 21 firm-year observations excluded seven samples that were not available in the Fn-Guide and three companies reported as duplicate companies in the one accounting period. Each continuous variable was winsorized at the 1st and 99th percentiles to minimize the effect of outliers.

### Event Study Model

To test our hypotheses, an event study method was used.^3^ Event studies are designed to measure the effect of an unanticipated event on stock prices. The day of the event (*t* = 0) is defined as the date when the company is reported to the media as a company in which an incident of bullying, and specifically a power trip has occurred. If there is no stock transaction for the company on the specified date, the first stock trading day after the media report is defined as the event date. The standard method is based on estimating a market model for each firm and then calculating abnormal returns. This study also uses a market model among the methods proposed by Brown and Warner (1985) to calculate the abnormal return (AR).^4^ These ARs are assumed to reflect the stock market’s reaction to the arrival of new information.

The method is as follows: The rate of return on the share price of firm *i* on day *t* is expressed as

(1) $$R_{i,t}=\alpha_{i}+\beta_{i}R_{m,t}+\varepsilon$$

where R_i,t_ is the rate of return on the share price of firm *i* on day *t*, and R_m,t_ is the rate of return on a market portfolio of stocks (such as the KOSPI index) on day *t*. In this study, α and β of equation (1) were estimated using the KOSPI equally weighted index (EWI) and the daily stock return on each firm.

We estimated the market model over 160 trading days, starting 5 days prior to the event day.^5^ Estimated α and β are used to measure the excess return (AR) of each company from day -5 to day +5. The method is as follows in equation (2).

(2) $$AR_{i,t}=R_{i,t}-{\hat{R}}_{i,t}=R_{i,t}-({\hat{\alpha }}_{i}+{\hat{\beta }}_{i}R_{m,t})$$

where $\hat{\alpha }$ and $\hat{\beta }$ are the ordinary least squares (OLS) parameter estimates obtained from the regression (1). R_i,t_ is the rate of return on the share price of firm *i* on day *t*, and R_m,t_ is the rate of KOSPI market return on day *t*.

After calculating the abnormal returns of individual firms using the market model, the following equation (3) was used to calculate the average abnormal return (AAR) for the entire firm reported by the media to have experienced an incidence of bullying or a power trip.

(3) $$AAR_{i,t}=\sum_{i=1}^{N}AR_{i,t}\times \frac{1}{N}$$

where N is the number of events being studied among multiple firms, AR is the abnormal return of firm *i* in period *t*, and AAR is the average abnormal return in period *t*.

In equation (3), the AARs are calculated. The cumulative average abnormal return (CAR) is calculated as follows when the AAR is accumulated during the event window. Using CAR, it is possible to analyze how the firm’s harassment behavior affects individual stock prices.

(4) $$CAR_{N}(t_{1},\;t_{2})=\sum_{t=t_{1}}^{t_{2}}ARR_{t}$$

where t_1_ and t_2_ are the initial and final dates of the event window, AAR is the average abnormal return in period *t*, and CAR is the cumulative AAR in period *t*.

### Regression Analysis Model

Hypotheses 1, 2, and 3 are verified by setting equations (5), (6), and (7) with the cumulative abnormal return before and after the event day as dependent variables. The method of using the CAR after the day before the event day has the advantage of mitigating the bias that may arise when analyzing only the return on the event day (*t* = 0). We set CAR as a dependent variable for 3 days from -1 day before the event day to 1 day after the event day. The cumulative abnormal returns over 3 days before and after the event day are a universal measure of stock returns used in event studies.

(5) $$\begin{array}{lll} CAR{\left(-1,1\right)}_{i,t} & = & \alpha_{0}+\beta_{1}SPDUM_{i,t}+\beta_{2}SIZE_{i,t}+\beta_{3}LEV_{i,t}\\ & & +\beta_{4}OCF_{i,t}+\beta_{5}ROA_{i,t}+\beta_{6}GROWTH_{i,t}\\ & & +\varepsilon \end{array}$$

(6) $$\begin{array}{lll} CAR{\left(-1,1\right)}_{i,t} & = & \alpha_{0}+\beta_{1}SPDUM_{i,t}+\beta_{2}SPDUM_{i,t}\\ & & \times DEMOTION_{i,t}+\beta_{3}SIZE_{i,t}+\beta_{4}LEV_{i,t}\\ & & +\beta_{5}OCF_{i,t}+\beta_{6}ROA_{i,t}+\beta_{7}GROWTH_{i,t}\\ & & +\varepsilon \end{array}$$

(7) $$\begin{array}{lll} CAR{\left(-1,1\right)}_{i,t} & = & \alpha_{0}+\beta_{1}SPDUM_{i,t}+\beta_{2}SPDUM_{i,t}\\ & & \times LARGE30_{i,t}+\beta_{3}LARGE30_{i,t}+\beta_{4}SIZE_{i,t}\\ & & +\beta_{5}LEV_{i,t}+\beta_{6}OCF_{i,t}+\beta_{7}ROA_{i,t}\\ & & +\beta_{8}GROWTH_{i,t}\mathrm{+\varepsilon} \end{array}$$

where CAR (-1,1) is the cumulative abnormal returns over 3 days from -1 day before the event day to 1 day after the event day. SPDUM is a dummy variable equal to 1 if companies were reported to media as firms charged with harassment, and 0 otherwise. DEMOTION is a dummy variable equal to 1 if companies were reported to the media as guilty of emotional harassment, and 0 otherwise. LARGE30 is a dummy variable equal to 1 if companies are conglomerate firm groups, and 0 otherwise. SIZE is computed as the natural logarithm of the total assets of the firm. LEV is defined as liabilities over equity. OCF is the operating cash flow scaled by lagged total assets. Return on assets (ROA) is net income divided by lagged total assets and GROWTH is growth of sales in year *t*.

Equation (5) is a model designed to test hypothesis 1 that suggests that the firm-level power trip has a negative effect on the firm’s value in the market. The firm’s harassment refers to the unfair conduct of an opponent who is in a favorable position in the firm. If the firm’s harassment is perceived as bad news in the capital market, the firm’s stock price will fall. Hypothesis 1 is supported if the coefficient β1 on the experimental variable SPDUM shows a significantly negative value.

The firm’s harassment behavior can be divided into emotional and non-emotional harassment. Equation (6) is a model designed to test hypothesis 2, which suggests the capital market’s response to the firm’s harassment is different for emotional harassment. In equation (5), the experimental variable is interaction term (SPDUM × DEMOTION). DEMOTION is a dummy variable equal to 1 if companies were reported to media as guilty of emotional harassment, and 0 otherwise.^6^ Hypothesis 2 is supported if the coefficient β2 on the interaction term (SPDUM × DEMOTION) shows a significantly negative value.

Equation (7) is a model designed to test hypothesis 3, which suggests the capital market’s response to the firm’s harassment is different if it is a conglomerate firm. In equation (7), the experimental variable is interaction term (SPDUM × LARGE30).^7^ Hypothesis 3 is supported if the coefficient β3 for the interaction term (SPDUM × LARGE30) shows a significantly negative value.

When the number of samples is small, reliability may be questioned in regression analysis. Therefore, the propensity score matching (PSM) method is used for analysis. In this study, a “1: 5” matching was conducted based on the media reports of the firm’s harassment, and OLS regression analysis was performed on a total of 126 firm-year observations.

The control variables of this study are as follows. SIZE is added to control the firm’s size. The larger the firm’s size, the more information there is about the company in the capital market (Fama and MacBeth, 1973). LEV is defined as liabilities over equity. In the capital market, the higher the liability, the higher the firm’s risk. The risk due to the debt ratio is added as a control variable because it may affect the ARs. Companies with a high cash flow ratio in operating activities are more likely to be affected by bad news because they are recognized as having superior ability to respond to crises. The ROA is added to control the impact of an individual firm’s performance on the excess return on the capital market. The better the company’s performance, the more likely it is that the company will achieve high excess returns in the capital market. GROWTH was included to control the growth of the enterprise.

## Empirical Results

### Market Reaction to Firm’s Harassment

**FIGURE 1:**
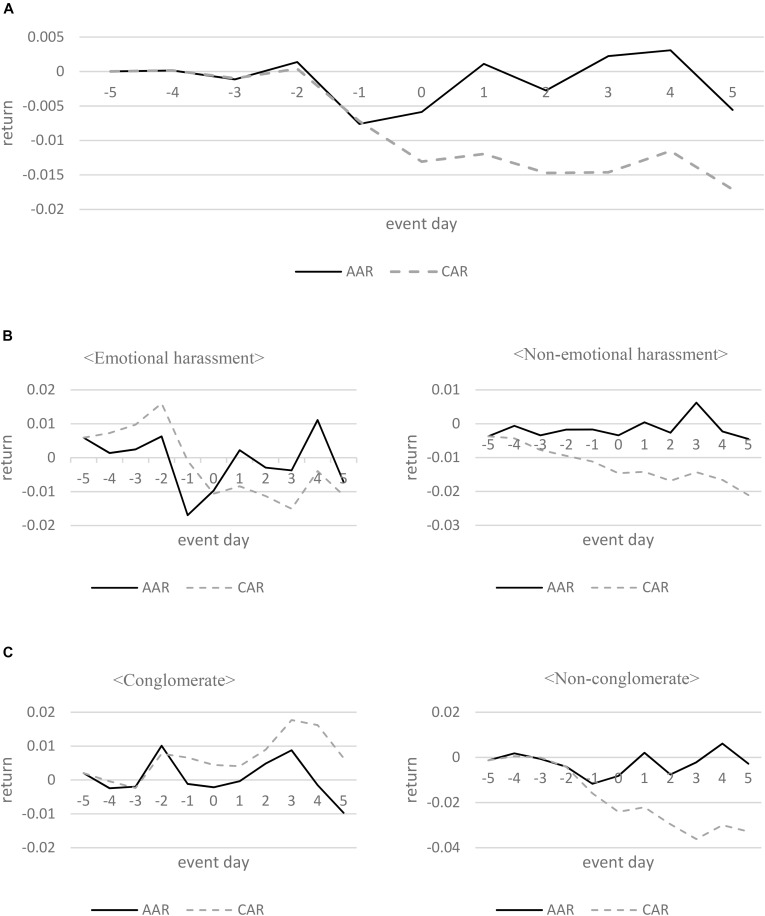
Abnormal and cumulative returns in the interval (–5, +5). **(A)** Abnormal returns of stocks resulting from a power trip. **(B)** Abnormal returns of stocks resulting from emotional and non-emotional harassment. **(C)** Abnormal returns of stocks in conglomerate and non-conglomerate firms.

Figure 1A shows the average abnormal return (AAR) and cumulative average excess return rate (CAR) from -5 to +5 days. The AAR is showing a sharp decline from -1 to +1 days, and CAR is beginning to decrease from -1 day before the event day but is showing a trend of repeating ups and downs.

Specifically, the AAR was -0.008 (*t* = -1.87, *p*-value = 0.071) at -1 day before the event day and -0.006 (*t* = -2.12, *p*-value = 0.042) at event day. CAR shows negative value from -1 day before the event day but shows a tendency to fall sharply at -0.013 (*t* = -1.68, *p*-value = 0.104) at event day. This result supports hypothesis 1 that the firm-level power trip has negative effects on a firm’s value in the stock market.

Figure 1B is the result of analyzing AAR and CAR before and after the event day after classifying the sample as either an emotional harassment group or a non-emotional harassment group. The first column in Figure 1B is the analysis of the emotional harassment group (*N* = 12) and the second column is the analysis of the non-emotional harassment group (*N* = 19).

According to the results of the analysis, the drop in AAR is relatively increased at the event day in the emotional harassment group. Specifically, the AAR of the emotional harassment group was -0.017 (*t* = -1.97, *p*-value = 0.074) at -1 day before the event day, -0.010 (*t* = -1.70, *p*-value = 0.117) at event day. While the AAR of the non-emotional harassment group was -0.002 at -1 day before the event day and -0.003 at event day, but there was no statistical significance found. These results are interpreted to mean that the capital market responds differently if emotional harassment is reported.

Figure 1C is the result of analyzing AAR and CAR before and after the event day after classifying the sample into conglomerate firm groups and non-conglomerate firm groups according to the definition of a conglomerate firm. The first column in Figure 1C is the analysis of the conglomerate firm groups (*N* = 12) and the second column is the analysis of the non-conglomerate firm groups (*N* = 19).

While the non-conglomerate firm group was statistically significant in the AAR during the event window, the conglomerate firm group was not statistically significant in the AAR in the same period. Specifically, the AAR of the conglomerate firm group was -0.001 at -1 day before the event day and -0.002 at event day, but this was not statistically significant, while the AAR of the non-conglomerate firm group was -0.012 (*t* = -2.00, *p*-value = 0.061) at -1 day before the event day and -0.008 (*t* = -2.06, *p*-value = 0.054) at event day. These results suggest that the capital market responds differently to events in the type of conglomerate firms. Please refer to Appendix 1 for specific figures for the abnormal returns for windows surrounding the event day.

Table 2 shows the results of the *t*-test used to analyze the impact of the firm’s harassment on the capital market reaction by cumulative abnormal returns. The sample was analyzed by dividing it into emotional and non-emotional harassment groups, and conglomerate and non-conglomerate firm groups.

**Table 2:** Cumulative abnormal returns for windows surrounding the event day.

**Panel A: Results of dividing power trips into emotional and non-emotional harassment groups**						

Window	Power trip (*N* = 31)		Emotional harassment (*N* = 12)		Non-emotional harassment (*N* = 19)	
			
	CAR	*t*-value	CAR	*t*-value	CAR	*t*-value
CAR (0,0)	-0.006	-2.12^∗∗^	-0.010	-1.70	-0.003	-1.27
CAR (-1,1)	-0.012	-1.85^∗^	-0.024	-1.93^∗^	-0.005	-0.66
CAR (-2,2)	-0.014	-1.46	-0.021	-1.18	-0.009	-0.85

**Panel B: Results of dividing power trips into conglomerate and non-conglomerate groups**						

**Window**	**Power trip (*N* = 31)**		**Conglomerate (*N* = 12)**		**Non-conglomerate (*N* = 19)**	
			
	**CAR**	***t*-value**	**CAR**	***t*-value**	**CAR**	***t*-value**

CAR (0,0)	-0.006	-2.12^∗∗^	-0.002	-0.66	-0.008	-2.06^∗^
CAR (-1,1)	-0.012	-1.85^∗^	-0.004	-0.48	-0.018	-1.84^∗^
CAR (-2,2)	-0.014	-1.46	0.011	0.81	-0.030	-2.59^∗∗^


*Variable definitions: AAR, the average abnormal return in period *t*; CAR, the cumulative average abnormal return in period *t*^∗^, ^∗∗^, ^∗∗∗^ indicate significance at the 10, 5, and 1% levels, respectively (two-tailed).*

The cumulative abnormal returns from the firm’s reported harassment show -0.006 (*t* = -2.12, *p*-value = 0.042) in CAR (0,0) and -0.012 (*t* = -1.85, *p*-value = 0.074) in CAR (-1,1), indicating that they were significant during the relatively short-term verification period. The results of dividing the sample into emotional and non-emotional harassment groups are as follows. In the emotional harassment group sample, the cumulative abnormal returns were -0.024 (*t* = -1.93, *p*-value = 0.080). Statistical significance was found only in this group.

The results of dividing the sample into conglomerate and non-conglomerate firm groups are as follows. In the non-conglomerate firms, the CARs are significantly negative during the entire verification period. However, there was no statistical significance found for CAR in conglomerate firms. This means that in non-conglomerate firms, CAR continued to decline to negative values after the event day.

The event study method is useful for directly analyzing the impact of specific information on the short-term capital market by excluding the influence of other factors, other than the experimental variables, on the stock price. However, the method is limited by the fact that it excludes corporate characteristics that could affect the firm’s abnormal returns. In this study, we conducted additional OLS regression analysis to include the financial factors that affect the firm’s abnormal return.

### Descriptive Statistics and Correlations

In this study, an OLS regression analysis was performed after constructing a “1: 5” control group by propensity score matching analysis. Table 3 provides descriptive statistics for the variables used in the regression analysis, while Table 4 shows the results of the analysis of difference of the major variables according to the firm’s harassment.

**Table 3:** Descriptive statistics.

	*N*	Mean	Std.	Min	Median	Max
**Panel A: Full sample**						
CAR (-1,1)	126	0.002	0.035	-0.101	-0.001	0.110
SPDUM	126	0.167	0.374	0	0	1
DEMOTION	126	0.071	0.259	0	0	1
LARGE30	126	0.571	0.497	0	1	1
SIZE	126	29.303	1.505	26.936	29.614	31.196
LEV	126	1.244	1.846	0.035	0.656	12.447
OCF	126	0.073	0.057	-0.019	0.070	0.220
ROA	126	0.030	0.044	-0.085	0.022	0.141
GROWTH	126	-0.004	0.131	-0.323	-0.007	0.333
**Panel B: Treatment firms (SPDUM = 1)**						
CAR (-1,1)	21	-0.011	0.041	-0.101	0.001	0.070
DEMOTION	21	0.429	0.507	0	0	1
LARGE30	21	0.571	0.507	0	1	1
SIZE	21	29.345	1.515	27.065	29.643	31.196
LEV	21	1.391	2.157	0.035	0.676	9.820
OCF	21	0.073	0.057	-0.012	0.070	0.220
ROA	21	0.026	0.042	-0.046	0.014	0.102
GROWTH	21	0.002	0.126	-0.323	-0.005	0.333
**Panel B: Control firms (SPDUM = 0)**						
CAR (-1,1)	105	0.004	0.034	-0.073	-0.002	0.110
DEMOTION	105	0	0	0	0	0
LARGE30	105	0.571	0.494	0	1	1
SIZE	105	29.294	1.510	26.936	29.584	31.196
LEV	105	1.215	1.787	0.035	0.656	12.447
OCF	105	0.073	0.058	-0.019	0.070	0.220
ROA	105	0.030	0.045	-0.085	0.022	0.141
GROWTH	105	-0.005	0.132	-0.323	-0.008	0.333


*Variable definitions: CAR (-1,1), cumulative abnormal returns over 3 days from -1 day before the event day to 1 day after the event day; SPDUM, a dummy variable equal to 1 if companies were reported to media as harassment firms, and 0 otherwise; DEMOTION, a dummy variable equal to 1 if companies were reported to media as emotional harassment, and 0 otherwise; LARGE30, a dummy variable equal to 1 if companies are conglomerate firm group, and 0 otherwise; SIZE, the natural logarithm of the total assets of the firm; LEV, liabilities over equity in year t; OCF, the operating cash flow scaled by lagged total assets; ROA, net income divided by lagged total assets; GROWTH, growth of sales in year *t*.*

**Table 4:** Differential analysis results for PSM matching firms.

Variables	Treatment firms (SPDUM = 1)			Control firms (SPDUM = 0)			Mean difference *t*-value	Wilcoxon *z*-value
				
	*N*	Mean	Median	*N*	Mean	Median		
CAR (-1,1)	21	-0.011	0.001	105	0.004	-0.002	-1.83^*^	-1.20
DEMOTION	21	0.429	0	105	0	0	8.80^***^	6.93^***^
LARGE30	21	0.571	1	105	0.571	1	0.00	0.00
SIZE	21	29.345	29.643	105	29.294	29.584	0.14	0.13
LEV	21	1.391	0.676	105	1.215	0.656	0.40	0.39
OCF	21	0.073	0.070	105	0.073	0.070	-0.02	0.05
ROA	21	0.026	0.014	105	0.030	0.022	-0.37	-0.47
GROWTH	21	0.002	-0.005	105	-0.005	-0.008	0.19	0.24


*See Table 3 for variable definitions. ^∗^, ^∗∗^, ^∗∗∗^ indicate significance at the 10, 5, and 1% levels, respectively (two-tailed).*

The standard variable of the difference analysis, SPDUM, is a dummy variable having a value of 1 if companies were reported by the media as harassment firms, or 0 otherwise. The mean value of SPDUM was 0.167. In other words, 21 firm-year observations of the full sample were firms reported by the media to have engaged in harassment.

Panel A of Table 3 presents the descriptive statistics for the entire sample, Panel B shows the descriptive statistics for the harassment firm (SPDUM = 1) group, and Panel C shows the descriptive statistics for the non-harassment firm (SPDUM = 0) group. In the entire sample, the dependent variable, CAR, was 0.002, which is not significantly different from 0, but the CAR of the SPDUM = 1 group is -0.011, which was lower than the CAR of the SPDUM = 0 group. This result supports hypothesis 1: the abnormal return of the companies reported by media to engage in harassment is low.

In the SPDUM = 1 group, the mean value of the DEMOTION was 0.429. In other words, 9 (= 21 × 0.429) companies were reported to the press as engaging in emotional harassment. LARGE30, the conglomerate firm group, was 0.571 in both SPDUM = 1 and SPDUM = 0 groups.

The mean (median) value of SIZE in the entire sample was 29.303 (29.614). The mean value of the debt-to-equity ratio (LEV) was 1.244, which reflects a sample of firms that are relatively sound financially. The mean (median) value of the operating cash flow (OCF) was 0.073 (0.070) and the mean (median) value of ROA was 0.030 (0.022). The mean (median) value of GROWTH, or the growth potential of the company, was -0.004 (-0.007).

Table 4 shows that there is no statistically significant difference between the treatment group and the control group in firm size (SIZE), debt ratio (LEV), operating cash flow (OCF), return on assets (ROA), or growth rate of sales (GROWTH). This is because when the propensity score matching method is considered, logit analysis is performed with the firm size, debt ratio, operating cash flow, total assets profit rate, and sales growth rate. In other words, the absence of statistical significance between the control variables in the analysis of difference means that the control group was appropriately selected. In that analysis, the mean value of CAR in the treatment group (-0.011) was significantly lower than the mean value of CAR in the control group (0.004).

Table 5 presents Pearson correlations among variables used in the main analyses. The correlation of SPDUM and CAR is negatively significant at 5%. The negative correlation between SPDUM and CAR suggests that a company reported to the media as engaging in power trips is associated with a negative stock return. There is a significant negative correlation between DEMOTION, which indicates whether the power trip involves emotional harassment, and CAR. Therefore, the first hypothesis gains support from the negative correlation between SPDUM and CAR.

**Table 5:** Pearson correlations.

Variables	CAR (-1,1)	SPDUM	DEMOTION	LARGE30	SIZE	LEV	OCF	ROA
SPDUM	-**0**.**162**							
DEMOTION	-**0**.**198**	**0**.**620**						
LARGE30	0.024	-0.000	-0.009					
SIZE	0.097	0.013	-0.023	**0**.**588**				
LEV	**0**.**183**	0.036	0.137	0.112	0.146			
OCF	0.043	-0.002	-0.020	-0.111	-0.070	-0.116		
ROA	-0.030	-0.033	-0.072	-**0**.**414**	-**0**.**449**	-**0**.**456**	**0**.**196**	
GROWTH	0.059	0.017	-0.010	-0.065	0.085	0.107	**0**.**598**	**0**.**233**


*See Table 3 for variable definitions; coefficients shown in bold are significant at *p* < 0.05 (two-tailed test).*

Also, the negative correlation between DEMOTION and CAR supports the second hypothesis which suggests that emotional harassment rather than non-emotional harassment will show more negative market reactions. The correlation between LARGE30 and SIZE is 0.59 has high positive significance in this regard. Among control variables, we find that few correlations are very high. For example, the correlation between OCF and GROWTH is 0.60, while that between LEV and ROA is -0.46. The variance inflation factor (VIF) for independent variables is 2.538 less than 10, which means that there is not a serious multi-collinearity problem.

## Regression Results

### Results for Market Reaction of Firm-Level Power Trips (H1)

Table 6 presents the results for the test of our first hypothesis from the regression analysis based on equation (5). In Table 6, hypothesis 1 is supported if coefficient β_1_ shows a significantly negative value. The results of the analysis show that the regression coefficient of SPDUM was -0.016 (*t* = -1.89, *p*-value = 0.061). These results suggest that participants in the capital market negatively perceive the press reports on the firm’s harassment behavior.

**Table 6:** Market reaction of firm-level power trip.

		CAR (-1,1)	
	
Variables	Exp.sign	Coef.	*t*-value
Intercept		-0.102	-1.37
SPDUM	–	-0.016	-1.89^*^
SIZE		0.003	1.31
LEV		0.005	2.33^**^
OCF		0.057	0.80
ROA		0.106	1.11
GROWTH		-0.017	-0.51
Adj. *R*^2^		0.035	
*N*		126	


*See Table 3 for variable definitions. ^∗^, ^∗∗^, ^∗∗∗^ indicate significance at the 10, 5, and 1% levels, respectively (two-tailed).*

### Results for Market Reaction by Emotional Harassment (H2)

Table 7 presents the results for the test of our second hypothesis from the regression analysis based on equation (6). In Table 7, hypothesis 2 is supported if coefficient β_2_ in the interaction term (SPDUM × DEMOTION) shows a significantly negative value. The result of the analysis shows that the regression coefficient of SPDUM × DEMOTION was -0.026 (*t* = -1.70, *p*-value = 0.093). This result implies that capital market participants perceive media reports on emotional harassment as more negative than media reports on non-emotional harassment.

**Table 7:** Market reaction by emotional harassment.

		CAR (-1,1)	
	
Variables	Exp.sign	Coef.	*t*-value
Intercept		-0.096	-1.30
SPDUM (β_1_)	–	-0.005	-0.44
SPDUM × DEMOTION (β_2_)	–	-0.026	-1.70^*^
SIZE		0.003	1.22
LEV		0.005	2.55^**^
OCF		0.059	0.83
ROA		0.104	1.10
GROWTH		-0.019	-0.57
Adj. R^2^		0.050	
*N*		126	
Test: β_1_ + β_2_ = 0 *F*-value (*p*-value)		6.46 (0.012)^∗∗^	


*See Table 3 for variable definitions. ^∗^, ^∗∗^, ^∗∗∗^ indicate significance at the 10, 5, and 1% levels, respectively (two-tailed).*

### Results for Market Reaction by Conglomerate (H3)

Table 8 presents the results for the test of our third hypothesis from the regression analysis based on equation (7). SPDUM is a regression coefficient that indicates the capital market response of media reports on a firm’s harassment behavior in non-conglomerate firms (LARGE30 = 0). The regression coefficient on SPDUM is -0.025 (*t* = -1.98, *p*-value = 0.050). In the non-conglomerate firm group, if the firm’s harassment behavior is reported by the media, it indicates that the company will have negative ARs. In hypothesis 3, we expect that the information use’s negative response in the conglomerate firm group would be greater than the information use’s negative response in the non-conglomerate firm group if the firm’s harassment behavior is reported by the media. The regression coefficient of SPDUM × LARGE30, which is the experimental variable of hypothesis 3, is 0.017 (*t* = 0.98, *p*-value = 0.331), which is not significant. In the conglomerate firm group, if the firm’s harassment behavior is reported by the media, there is no effect on ARs. The sum of the regression coefficients of SPDUM and SPDUM × LARGE30 was -0.008 (= -0.025 + 0.017), which was not statistically significant.

**Table 8:** Market reaction by conglomerate.

		CAR (-1,1)	
	
Variables	Exp.sign	Coef.	*t*-value
Intercept		-0.110	-1.33
SPDUM (β_1_)	–	-0.025	-1.98^**^
SPDUM × LARGE30 (β_2_)	–	0.017	0.98
LARGE30	–	-0.005	-0.62
SIZE		0.004	1.28
LEV		0.005	2.26^**^
OCF		0.055	0.78
ROA		0.099	1.02
GROWTH		-0.016	-0.49
Adj. R^2^		0.027	
*N*		126	
Test: β_1_ + β_2_ = 0 *F*-value (*p*-value)		0.62 (0.432)	


*See Table 3 for variable definitions. ^∗^, ^∗∗^, ^∗∗∗^ indicate significance at the 10, 5, and 1% levels, respectively (two-tailed).*

This result is not consistent with hypothesis 3 that the effect of a firm-level power trip on a firm’s value in the market is more salient for the conglomerate firms’ group than for the non-conglomerate firms’ group. These results reflect the perception of market participants that conglomerate firms will not fail because they enjoy a competitive edge in capital, labor, and products. In addition, the results of Table 8 are consistent with the results of the event study analysis in which the CAR variable of non-conglomerate firms is negative in all periods, while the CAR variable of conglomerate firms is not significant in Figure 1C.

## Discussion

This study examined the effects of power trips by top managers on the firm’s valuation in the market. We found that power trips revealed by the mass media have significant and negative effects on a firm’s value. We also found that this negative impact is moderated by the type of power trip. This study contributes to the literature in several ways. First, it complements the existing literature by explaining how markets evaluate unethical behavior by top managers. Although many studies have highlighted the malfunctioning and counterproductive workplace behaviors caused by power trips within the firm, few have explored the firm-level consequences of power trips. The issue of power trips should be considered at both the micro- (i.e., the individual and team level) and the macro-level (of the organization or firm). This study is an effort to fill gaps in the research by exploring the consumers’ and investors’ perspectives. Second, this study suggests the importance of emotion in the analyses of power trips based on emotional contagion and event system theory. The role of emotions in both the service encounters, where positive or negative emotions spread from employee to consumer, and in media coverage, where negative emotions spread from the emotional harassment event by TMTs to consumers, is still important. The event system theory assumes that events are not homogeneous but heterogeneous. The strength of an event evolves systemically in organizations across space and time. Events cannot be treated as discrete and isolated matters of concern at only one level. Thus, power trips are disruptive events that force organizations out of their conventional response modes. Prior research highlights how employees experience emotional events at work, while affective event theorists have considered how workplace events affect employees’ emotions (Weiss and Cropanzano, 1996; Weiss et al., 1999). In this study, we have explored how markets estimate the news of emotional harassment. Our finding is aligned with previous studies of leadership theories. Humphrey et al. (2016) emphasize the important role of emotional factors and represent the usefulness of merging research on leadership and emotions. Empirical studies have also shown that a leader’s emotional expressions are more important than the content of the message (Newcombe and Ashkanasy, 2002) because leaders influence follower’s attitudes, cognitions, affective states, and behavior (Koning and Van Kleef, 2015). In this vein, we suggest that emotional power tripping by leaders leads to detrimental consequences. Third, our research provides a unique hypothesis of power trips in which effects are contingent on the firm’s visibility. However, this hypothesis is not supported, and this can be explained by the following reason. As most conglomerate firms are not businesses that directly deal with consumers, the information that conglomerate firms are reported to the press as firms guilty of harassment may not significantly affect the market participants’ judgment on the future profitability of the company. On the other hand, non-conglomerate firms have many substitute goods and often deal directly with consumers. Therefore, if media reports precipitated by firms’ harassment behaviors (unethical behavior) are spread by consumer boycotts, they will directly impact the profitability of the company. Thus, the negative relationship between the firm’s harassment behaviors and abnormal returns is not apparent in the conglomerate firms, but the relationship for non-conglomerate firms is significantly negative. In fact, Namyang Co., which was reported to the media as a conglomerate firm guilty of harassment, reported a drop in operating profits by 87.7%.^8^ This evidence is consistent with Korean investors believing the very largest conglomerate firms are “too big to fail.” The advantageous position of conglomerates in Korean context is widely recognized in the literature from angles of government (Lee et al., 2002) and networking capabilities (Kim, 2005). This is also in consonance with Agarwal et al. (2002)’s study. Her empirical work demonstrates that largeness lowers mortality rates by improving firms’ ability to shield themselves from uncertain environments. And the idea of liability of smallness indicates that mortality rates decline with increased size (Hannan and Freeman, 1984). Empirical regularities, in economics, also show a positive relationship between firm size and survival for any given growth rate (Sutton, 1997). This is because the benefits of greater market power (Bain, 1956) and the minimum efficient scale (Mansfield, 1962; Jovanovic, 1982). Thus, the idea that bigger is better contributes to the lower failure rate among conglomerates.

The study has limitations. The empirical analysis was based on listed firms in a single country—Korea—and therefore its generalizability may be limited. The unlisted business in an informal economy shows different behaviors with respect to power trips and thus the consequences will not be consistent with those of listed firms. The causes and outcomes of power tripping can also be found in the cultural context, the social climate, and in psychological factors. According to Hofstede’s cultural distance index, Korean culture is known for its high-power distance, high uncertainty avoidance, and short-termism. The Korean decision-making process is also heavily influenced by hierarchies. Such a cultural context makes Korean society a place conducive to power trips because people can easily draw on superior power to justify unethical or aggressive behaviors. In this regard, future research could provide valuable insights into the embeddedness of firm behaviors in various cultural and institutional environments.

## Author Contributions

Sj-C devised the project, the main conceptual ideas, and was in charge of overall direction and planning. YC developed the theoretical framework and took the lead in writing the manuscript. HP contributed to the design and implementation of the research and to the analysis of the results.

## Conflict of Interest Statement

The authors declare that the research was conducted in the absence of any commercial or financial relationships that could be construed as a potential conflict of interest.

## Footnotes

1 Korean Air Vice President Cho Hyun-ah was charged with violating aviation safety law. Ms. Cho also forced the flight crew and the junior attendant who served the nuts to apologize on their knees like slaves. She used abusive language and threw documents. Her bullying undoubtedly affected many important relationships at Korean Air. Because she is the daughter of Cho Yang-ho, the airline’s chairman, it is likely that when the prosecution’s investigation began, the share price, which had soared to 19% before the incident, plummeted by more than 200 billion won (an organization-level feature) (https://www.nytimes.com/2018/04/13/world/asia/nut-rage-sister-korean-air.html)

2 In the example above, Korean Air is a conglomerate firm. On December 5, 2014, the incident occurred in a case where the person uttered a speech with a profanity. Therefore, the LARGE30 variable is 1 and the DEMOTION variable is 1. On the other hand, Namyang Co., is a non-conglomerate firm. On May 8, 2013, the incident occurred due to forced sales to subcontractors. Therefore, the LARGE30 variable is 0 and the DEMOTION variable is 0.

3 The event study method in accounting has been used in numerous empirical studies since Fama et al. (1969) analyzed the disclosure effect of stock splits for an event study. The first step in the event study is to define the events to be studied and determine the duration of the events. An event period is a period of time when specific information is considered to have an impact on the stock price. In general, the event period is set up according to the event date of the specific information, and includes the before and after periods. The reason for including the pre-event date in the event period is that there is a possibility that the specific information is known to the market before the event date. On the other hand, the reason for inclusion in the event period after the event date is to take into consideration the case where information is disclosed after the stock market is closed.

4 Brown and Warner (1980, 1985) found that the constant mean-return model performs as well as more sophisticated approaches.

5 In the previous studies (Bruner and Simms, 1987; Rutherford, 1990; Dickinson et al., 1991; Agrawal and Kamakura, 1995), the analysis was conducted using 160 trading days.

6 The firm’s harassment behavior is revealed to the capital market through the media (e.g., the Internet, news, etc.). In addition, we analyze whether the emotional harassment occurred by analyzing the reported media. The DEMOTION variable was not included in equation (6) because it cannot distinguish whether the emotional harassment that is not reported in the press is indicative of a firm’s harassment behavior.

7 Conglomerate firms are often called *chaebol* firms. The definition used to identify *chaebol* firms is that of a large business group established by the Korea Fair Trade Commission (KFTC) of which more than 30% of the shares are owned by the group’s controlling shareholders and its affiliated companies.

8 In May 2013, Namyang Co., was criticized following an accusation that, for a long time, the firm had strongly pressed local franchises to buy its products, as well as a tape-recording of rough words to local owners. Namyang Co., caused more than about 18 million dollars as of July 2018 of damage through illegal activities such as high-pressure sales and shifting wages of salesmen to local franchises for the past seven years. Angry over the behavior at Namyang Co., consumers started to boycott its products.

## APPENDIX 1

**Appendix 1:** Abnormal returns for windows surrounding the event day.

**Panel A: Abnormal returns of stocks concerning reported power trip**								

**Event day**	**Power trip (*N* = 31)**							
	
	**AAR**		***t*-value**		**CAR**		***t*-value**	
-5	0.000		0.01		0.000		0.01	
-4	0.000		0.04		0.000		0.03	
-3	-0.001		-0.27		-0.001		-0.17	
-2	0.001		0.35		0.000		0.06	
-1	-0.008		-1.87^*^		-0.007		-1.00	
0	-0.006		-2.12^**^		-0.013		-1.68	
1	0.001		0.24		-0.012		-1.20	
2	-0.003		-0.82		-0.015		-1.31	
3	0.002		0.60		-0.015		-1.13	
4	0.003		0.93		-0.012		-0.92	
5	-0.006		-1.71^*^		-0.017		-1.46	

**Panel B: Abnormal returns of stocks on emotional harassment and non-emotional harassment**								

**Event day**	**Emotional harassment (*N* = 12)**				**Non-emotional harassment (*N* = 19)**			
		
	**AAR**	***t*-value**	**CAR**	***t*-value**	**AAR**	***t*-value**	**CAR**	***t*-value**

-5	0.006	0.85	0.006	0.85	-0.004	-1.48	-0.004	-1.48
-4	0.001	0.20	0.007	0.65	-0.001	-0.16	-0.004	-0.93
-3	0.002	0.27	0.010	0.98	-0.003	-0.85	-0.008	-1.14
-2	0.006	1.42	0.016	1.39	-0.002	-0.30	-0.009	-1.48
-1	-0.017	-1.97^*^	-0.001	-0.06	-0.002	-0.51	-0.011	-1.47
0	-0.010	-1.70	-0.011	-0.68	-0.003	-1.27	-0.015	-1.73
1	0.002	0.43	-0.008	-0.47	0.000	0.06	-0.014	-1.19
2	-0.003	-0.42	-0.011	-0.50	-0.003	-0.77	-0.017	-1.40
3	-0.004	-0.58	-0.015	-0.60	0.006	1.38	-0.014	-1.02
4	0.011	1.72	-0.004	-0.16	-0.002	-0.75	-0.017	-1.29
5	-0.007	-1.39	-0.011	-0.47	-0.004	-1.05	-0.021	-1.79^*^

**Panel C: Abnormal returns of stocks on conglomerate and non-conglomerate firms**								

**Event day**	**Conglomerate (*N* = 12)**				**Non-conglomerate (*N* = 19)**			
		
	**AAR**	***t*-value**	**CAR**	***t*-value**	**AAR**	***t*-value**	**CAR**	***t*-value**

-5	0.002	0.42	0.002	0.42	-0.001	-0.30	-0.001	-0.30
-4	-0.002	-0.37	-0.000	-0.04	0.002	0.44	0.001	0.10
-3	-0.002	-0.38	-0.002	-0.23	-0.001	-0.10	-0.000	-0.01
-2	0.010	1.36	0.008	0.62	-0.004	-1.05	-0.004	-0.64
-1	-0.001	-0.25	0.007	0.49	-0.012	-2.00^*^	-0.016	-2.04^*^
0	-0.002	-0.66	0.004	0.36	-0.008	-2.06^*^	-0.024	-2.54^**^
1	-0.000	-0.05	0.004	0.23	0.002	0.34	-0.022	-1.93^*^
2	0.005	0.79	0.009	0.45	-0.008	-2.13	-0.030	-2.37^**^
3	0.009	1.46	0.018	0.79	-0.002	-0.45	-0.036	-2.65^**^
4	-0.002	-0.30	0.016	0.69	0.006	1.40	-0.030	-2.37^**^
5	-0.010	-3.07^**^	0.006	0.29	-0.003	-0.57	-0.033	-2.86^**^

*Variable definitions: AAR, the average abnormal return in period *t*; CAR, the cumulative average abnormal return in period *t*; ^∗^, ^∗∗^, ^∗∗∗^ indicate significance at the 10, 5, and 1% levels, respectively (two-tailed).*
